# A look at the past to draw lessons for the future: how the case of an urgent ICU transfer taught us to always be ready with a plan B

**DOI:** 10.3389/fmed.2023.1253673

**Published:** 2023-11-20

**Authors:** Laura Brunelli, Edoardo Miotto, Massimo Del Pin, Daniele Celotto, Adriana Moccia, Gianni Borghi, Amato De Monte, Cristiana Macor, Roberto Cocconi, Luca Lattuada, Silvio Brusaferro, Luca Arnoldo

**Affiliations:** ^1^Dipartimento di Area Medica, Università degli Studi di Udine, Udine, Italy; ^2^SOC Rischio Clinico, Qualità e Accreditamento, Azienda Sanitaria Universitaria Friuli Centrale, Udine, Italy; ^3^Direzione Medica, Presidio Ospedaliero Universitario S. Maria della Misericordia di Udine, Azienda Sanitaria Universitaria Friuli Centrale, Udine, Italy; ^4^Dipartimento di Anestesia e Rianimazione, Azienda Sanitaria Universitaria Friuli Centrale, Udine, Italy

**Keywords:** ICU transfer, risk management, healthcare-associated infections, emergency, preparedness

## Abstract

**Objective:**

The urgent transfer of an intensive care unit (ICU) is particularly challenging because it carries a high clinical and infectious risk and is a critical node in a hospital’s patient flow. In early 2017, exceptional rainfall damaged the roof of the tertiary hospital in Udine, necessitating the relocation of one of the three ICUs for six months. We decided to assess the impact of this transfer on quality of care and patient safety using a set of indicators, primarily considering the incidence of healthcare-associated infections (HAIs) and mortality rates.

**Methods:**

We performed a retrospective, observational analysis of structural, process, and outcome indicators comparing the pre- and posttransfer phases. Specifically, we analyzed data between July 2016 and June 2017 for the transferred ICU and examined mortality and the incidence of HAI.

**Results:**

Despite significant changes in structural and organizational aspects of the unit, no differences in mortality rates or cumulative incidence of HAIs were observed before/after transfer. We collected data for all 393 patients (133 women, 260 men) admitted to the ICU before (49.4%) and after transfer (50.6%). The mortality rate for 100 days in the ICU was 1.90 (34/1791) before and 2.88 (37/1258) after transfer (*p* = 0.063). The evaluation of the occurrence of at least one HAI included 304 patients (102 women and 202 men), as 89 of them were excluded due to a length of stay in the ICU of less than 48 h; again, there was no statistical difference between the two cumulative incidences (13.1% vs. 6.9%, *p* = 0.075).

**Conclusion:**

In the case studied, no adverse effects on patient outcomes were observed after urgent transfer of the injured ICU. The indicators used in this study may be an initial suggestion for further discussion.

## Introduction

The need for urgent transfer of an entire hospital unit due to an emergency that may be triggered by natural and human-induced hazards (1) is an event that can occur (2–4) and requires healthcare facilities to have preparedness plans for such critical situations (5). Although there are some studies in the scientific literature reporting the experience and outcomes of evacuations during disasters such as hurricanes (2, 3) and suggesting strategies for preparing and implementing effective evacuation of intensive care units (ICUs) during a disaster (4), there are still gaps in knowledge regarding the potential systemic and medium- or long-term impact on quality of care and patient safety when the destination of the transfer is the same hospital. Indeed, the most common situation is that all patients are transferred to another hospital (especially a highly specialized center) that is not affected by the same threat as the first one (6).

The functional and organizational location of the ICU between the emergency department, operating rooms, and normal wards makes the performance of these units highly interdependent and complex. The functioning of an ICU actively contributes to good patient management throughout the hospital by reducing waiting times for surgical and specialized medical services and curbing patient flow during admission and discharge (7). Patient care in the ICU differs from that in other wards in the intensity and closer interaction between healthcare professionals and patients and is characterized by a high level of technology and skilled personnel (8). To meet the high demands of the facility, skilled personnel, technology, and organization, the critical characteristics of these departments in terms of architecture, health staff, technology, and organization have been studied and informed in detail at the international (8–11) and national levels (12). Nevertheless, sound strategies have been developed to ensure a more pleasant work environment focused on staff well-being, which is now considered the fourth essential goal for quality of care in healthcare facilities (13, 14). Because the ICU is one of the units where the most severe and life-threatening illnesses and injuries are treated and where there is a high clinical and infectious risk, it is a care environment where the occurrence of human-induced hazards, such as structural damage (1), although not common, can have a very negative impact on patient outcomes.

One of the major clinical risks in ICUs is the occurrence of healthcare-associated infections (HAIs), which affected 8.3% of patients who spent more than 2 days in a European ICU in 2017, particularly pneumonia, bloodstream infections (BSIs), and urinary tract infections (UTIs). Most of these infections were related to the invasive procedures of intubation, the insertion or care of vascular catheters, and the presence of bladder catheters (15). In addition, the ICU has been described as a stressful and highly demanding work environment associated with difficulties in work-life balance related to shift work, possibly leading to psychological problems among healthcare staff. Hall et al., in turn, found in their review that poor well-being (e.g., depression, anxiety, poor quality of life, stress) and moderate to high levels of burnout were associated with higher risk for patient safety through the occurrence of self-reported and objectively measured medical errors (16). As early as the 2000s, patient safety was also found to be negatively related to staff workload (17, 18). To date, few studies have described the impact of ICU transfer on the incidence of HAIs and the consequences for overall patient outcomes (19, 20).

The primary aim of this study was to evaluate the impact of a six-month temporary transfer of an ICU within the same hospital on the incidence of HAIs and mortality rates by comparing the 6 months period before the unplanned transfer with the 6 months period in the recovery room of the transferred ICU, considering the two indicators as the main outcomes. The secondary aim was to better understand the impact on quality of care and patient safety by developing and examining a more complete set of structural, process, and outcome indicators.

## Materials and methods

This paper reports an ex-post analysis of an adverse event that occurred in one of the intensive care units of our hospital in Udine in early 2017 and required its urgent transfer. Udine hospital is a 900-bed tertiary care hospital in northeastern Italy that serves a population of approximately 516,000 and has three intensive care units. Prior to the events, the hospital had 27 ICU beds: eight in ICU-A, eleven in ICU-B, and eight in ICU-C. Recognizing that organizational, environmental, and professional aspects together play a fundamental role in the ultimate determination of clinical outcomes for patients, we decided to examine the multilevel impact of the urgent transfer of an ICU in our hospital in early 2017 from the perspectives of risk assessment, risk management, and contingency planning. Due to the exceptional rainfall between January 6 and 8, the ICU-B experienced roof damage and flooding. To ensure the safety of patients and healthcare staff, ICU-B was urgently relocated from its original location to an alternate location in the recovery room of the operating rooms of another hospital building at night. This relocation ultimately lasted six months, until July 2017.

To examine the multilevel impact of the urgent ICU transfer at our hospital, we developed a set of indicators (Structural-S, Process-P, and Outcome-O) to conduct a retrospective, observational analysis of clinical and organizational outcomes.

S – ICU-B only. Structural indicators examined the structural and technical characteristics of ICU-B before and after transfer, including information such as the total area of the unit (m^2^), the number of single rooms, the number of windows, the presence of a teamwork area, the presence of a central monitoring system, and the presence of dedicated areas for procedures and medication preparation (8, 10, 21).

P – for all intensive care units. Process indicators included data on completeness of clinical records, use of alcohol-based hand sanitizers (22), number of patients admitted, length of stay in the ICU, and Diagnosis Related Group (DRG) type of all patients admitted to all ICUs in the hospital between 2015 and 2019 (7, 10, 21).

O – for all ICUs except HAIs. Outcome indicators included adverse events (23, 24), workplace violence (25), occupational injuries reporting, and patient complaints (26); condition at ICU discharge; mortality rates between 2015 and 2019, stratified by a six-month period for all hospital ICUs; for ICU-B, we assessed the incidence of HAIs among patients before and after transfer (7). We examined the incidence of HAIs using only ICU-B clinical records, including all patients admitted to ICU-B at Udine Hospital between July 1, 2016 and June 30, 2017. Clinical records were analyzed for inpatients who were older than 18 years and whose informed consent for data use for research purposes was registered in the hospital databases. Data included age, sex, situation before admission, date of ICU admission and discharge, condition at ICU discharge, SAPS II (Simplified Acute Physiology Score), start and end of invasive device use, and occurrence of HAI. If a patient was admitted to an ICU more than once during hospitalization, only the first admission was considered. The criteria of the European Center for Disease Prevention and Control (ECDC) were used to define the occurrence of HAI (12, 13), so we excluded patients who spent less than 48 h in the ICU for the assessment of HAI incidence. Data were collected anonymously and pooled by the research team for statistical analysis. Only data from patients for whom consent for data use for research and improvement purposes was on file in hospital records were included. The study protocol was approved by the Regional Unique Ethics Committee (CEUR) of Friuli-Venezia Giulia.

### Analysis of the data

To analyze the main outcome indicators (HAI and mortality rate) as a direct effect of transfer, ICU-B patients were divided into two groups: the pretransfer group, which included those admitted to ICU-B between July 1, 2016, and discharged by January 9, 2017, and the posttransfer group, which included those admitted to ICU-B from January 9 and discharged before June 30, 2017. We excluded patients who were transferred from a first ICU to a second ICU because their experiences would have differed from those of patients discharged before the adverse event and from those of patients discharged to the ICU during the posttransfer period.

To assess the overall impact on activity of all hospital ICUs, we examined 6 months mortality rates for 2015–2019 (January–June and July–December) and stratified them by DRG type (medical or surgical). Statistical analyses were performed using the Chi-square test, Kolmogorov–Smirnov test for normality, parametric (Student’s *t*-test), and nonparametric (Mann–Whitney) tests, with a value of *p* of <0.05 considered statistically significant. Analyses were performed using IBM SPSS 2019 Statistics software (IBM, Bologna, Italy).

## Results

### S – description of the structural indicators of ICU-B before and after transfer

Table 1 shows the structural characteristics of ICU-B at the original site and after relocation.

**Table 1:** Structural and technical characteristics of ICU-B before and after transfer.

Structural indicators for ICU-B		Before transfer	After transfer
Structural characteristics of the unit	Total area (m^2^)	228	150
	Number of beds	12	8
	Possible expansion of beds	+1 bed	0
	Number of single rooms	2	0
	Number of common rooms	2 (7 + 3 beds)	1 open room (8 beds)
	Number of windows	Minimum 1/bed	None
	Wall color	White	Blue
Technical equipment	Possibility to provide positive or negative pressure isolation	2 beds separated	Absent
	Technical equipment	Hanging on the ceiling	Hanging on the walls
	Continuous individual monitoring of vital sign	Available	Available
	Centralized system for monitoring vital signs	Available	Absent
	Room temperature control	Available	Available
Supplies	Meals, enteral and parenteral nutrition	Centralized hospital service	Centralized hospital service
	Storage room	Available	Available
Dedicated areas	Clinical support zone (e.g., for team interaction and central activities)	Available	Available
	Unit support zone (e.g., for handoffs, meetings, and ward administration)	Available	Absent
	Dedicated area for procedures	Available	Absent
	Dedicated area medications preparation	Available	Absent
Accessibility	Measures to limit access by outsiders	Available	Available
	Video entry monitoring system	Absent	Absent
	Family support zone (e.g., for talking with family members or visitors)	Available	Available
	Dedicated elevator of appropriate size	Available	Available
	Availability of vertical and horizontal connections to other hospital areas	Available	Available

### P – description of process organizational and clinical risk indicators before and after transfer

Regarding process indicators, the quality and completeness of clinical records after transfer showed an improvement for ICU-B (from 70.5% to 78.0%), while ICUs A and C, generally achieved higher scores but showed a minimal decrease (ICU-A from 86.0% to 85.0%, ICU-C from 93.0% to 91.0%). Regarding the consumption of alcohol-based hand sanitizers, considered here as a proxy indicator of hand hygiene compliance, consumption decreased in the post-transfer period in ICU-B (from 79.0 liters to 59.5 liters), while it increased in ICU-A (from 45.0 liters to 61.1 liters) and in ICU-C (from 61.9 liters to 83.5 liters). Regarding workplace violence and staff injuries, there were a total of eight episodes of workplace violence and injuries in the three ICUs before transfer and six after transfer. There were no changes in the number of patient complaints during the study period.

### O – description of patient outcome indicators before and after transfer

#### Impact of transfer on mortality in all ICUs

Table 2 shows data on the total number of patients admitted to the ICU, the number of days in the ICU, the number of patients who died in the ICU, the mortality rate, stratified by type of DRG (medical or surgical), and the mortality rate per 100 days in the ICU for each semester from 2015 to 2019. The semester of interest for ICU transfer (H1 2017) had three of the four highest rates for 2015–2019, with the mortality rate for the surgical Diagnosis Related Group being the second highest for that period.

**Table 2:** Output and outcome measures related to all three IUCs, stratified by semester, from 2015 to 2019.

	N. of patients	ICU hospitalization days	Deaths	Mortality %	Mortality per 100 days	DRG					
						Medical			Surgical		
						N.	Deaths	Mortality %	N.	Deaths	Mortality %
1° semester 2015	585	4,346	137	23.4	3.2	205	65	31.7	380	72	18.9
2° semester 2015	632	4,340	144	22.8	3.3	209	81	38.8	423	63	14.9
1° semester 2016	660	4,312	166	25.2	3.8	213	86	40.4	447	80	17.9
2° semester 2016	587	4,539	134	22.8	3.0	205	57	27.8	382	77	**20.2**
1° semester 2017	570	4,073	157	**27.5**	**3.9**	198	85	**42.9**	372	72	19.4
2° semester 2017	604	3,979	128	21.2	3.2	195	69	35.4	409	59	14.4
1° semester 2018	642	4,155	148	23.1	3.6	214	83	38.8	428	65	15.2
2° semester 2018	645	3,925	130	20.2	3.3	203	62	30.5	442	68	15.4
1° semester 2019	731	4,426	144	19.7	3.3	236	66	28.0	495	78	15.8
2° semester 2019	682	4,101	139	20.4	3.4	235	74	31.5	447	65	14.5

In “bold” the highest rate, “underlined” the lowest one.

The total number of adverse events in the hospital’s ICUs increased from 17 to 32, primarily due to an increase in reported adverse events in ICU-C (from 7 to 19), while the number of reports in ICUs A and B remained virtually unchanged. The number of adverse events reported by health professionals in the three ICUs also appeared to follow the same trend: it increased from 35 to 49, and this increase was also mainly due to ICU-C (from 10 to 28).

#### The impact of transfer on ICU-B

During the study period, a total of 394 patients were admitted to ICU-B, and one patient was excluded from the study because he was admitted to ICU-B twice (once per phase). Thus, we analyzed data from 393 patients, of whom 133 were women (33.8%) and 260 were men (66.2%). Specifically, 194 (49.4%) patients were admitted in the pretransfer period and 199 (50.6%) in the posttransfer period. Table 3 summarizes the main characteristics of patients admitted to ICU-B before and after transfer.

**Table 3:** Main characteristics of patients admitted to ICU-B for at least 48 h stratified by phase before and after transfer.

Variable	Before transfer (*N* = 160) n. (%)	After transfer (*N* = 144) n. (%)	Value of *p*
**Type of admission**			
Medical	88 (55.0)	75 (52.1)	0.407
Surgical-elective	27 (16.9)	33 (22.9)	
Surgical-urgent	45 (28.1)	36 (25.0)	
**Comorbidities**			
0–1	35 (21.9)	27 (18.8)	0.569
2 or more	125 (78.1)	117 (81.2)	
SAPS II (N = 160+*141)			
Less than 52	134 (83.8)	112 (79.4)*	0.333
52 or more	26 (16.2)	29 (20.6)*	
**Intubation**			
No	32 (20.0)	28 (19.4)	0.903
Yes	128 (80.0)	116 (80.6)	
**Central venous catheter**			
No	31 (19.4)	35 (24.3)	0.298
Yes	129 (80.6)	109 (75.7)	

*The number in brackets represents the patient sample size when not corresponding to the total study population.

The number of patients who died in ICU-B was 71 (cumulative incidence × 100 patients: 18.1%), in the pretransfer period 34 (17.5%), and in the posttransfer period 37 (18.6%); the mortality rate for 100 days in the ICU was 1.90 (34/1791) before transfer and 2.88 (37/1258) after transfer, with no statistically significant differences. There were also no significant differences in DRG type between the two periods; the number of DRGs with complications was 48 (24.7%) in the pretransfer phase and 56 (28.1%) in posttransfer phase, and analysis of DRG weights showed 2.61 ± 2.08 in the first phase and 2.86 ± 2.17 after transfer.

#### Focus on HAIs

**Figure 1:**
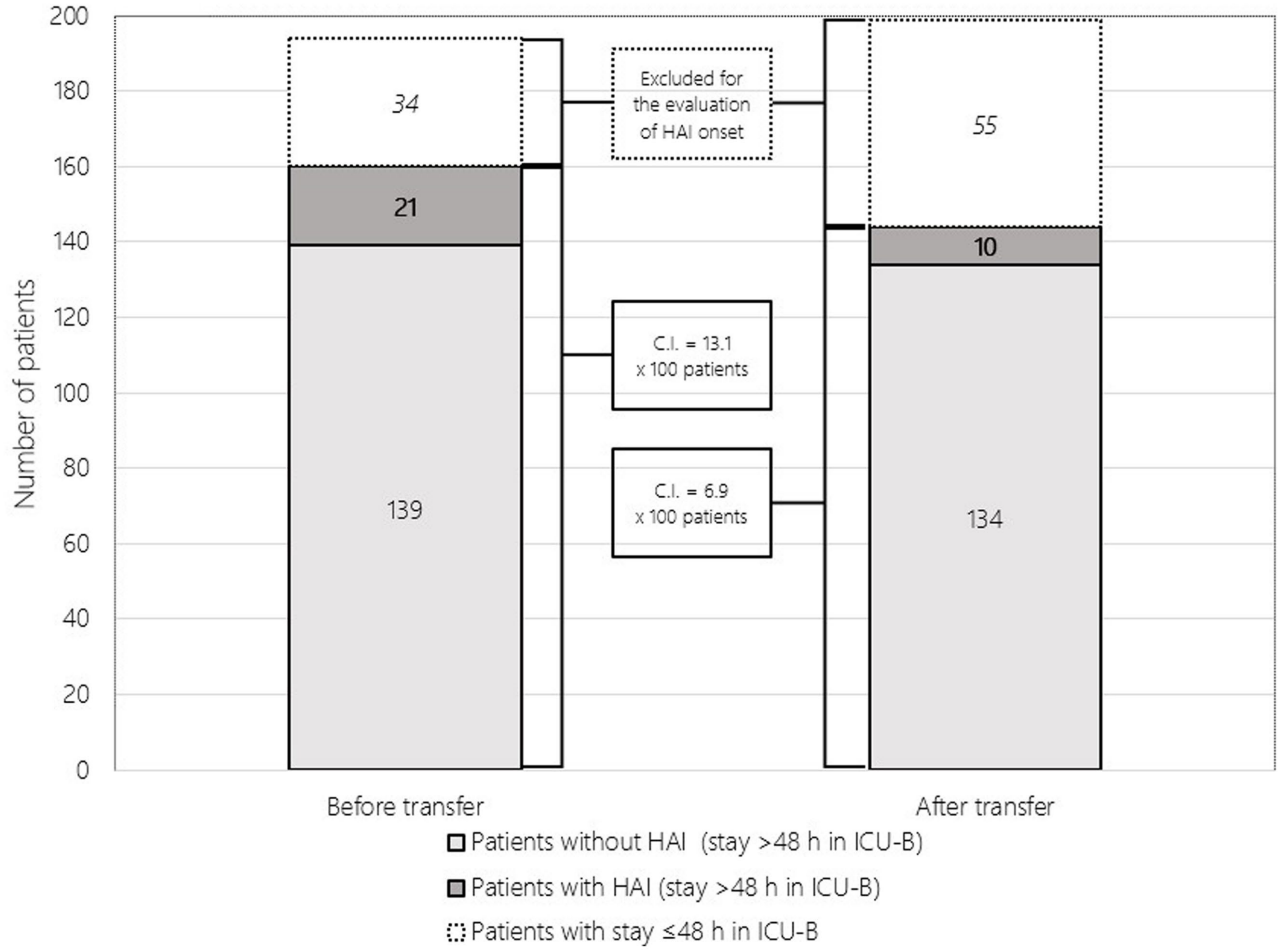
Patients in ICU-B stratified by phase (before and after transfer) and by subsample for assessment of HAI occurrence (stay >48 h). HAI, healthcare-associated infections; ICU-B, intensive care unit B; C.I., cumulative incidence (i.e., cumulative incidence of HAI during the study period).

The total number of patients included in the study of the occurrence of at least one HAI is shown in Figure 1.

The mean age ± SD of patients was 64.7 ± 15.4 years, 66.3 ± 13.6 years in women (n. 102; 33.6%) and 63.9 ± 16.3 years in men (n. 202; 66.4%). Before transfer, 160 patients were admitted with a mean age of 63.4 ± 16.7 years, including 53 women (33.1%) with 64.6 ± 14.9 years and 107 men (66.9%) with 62.7 ± 17.4 years. After transfer, 144 patients were admitted with a mean age of 66.2 ± 13.8 years, including 49 (34.0%) women with 68.1 ± 12.0 years and 95 (66.0%) men with 65.2 ± 14.6 years. There were no statistical differences between the two phases when comparing age and sex. Patients before transfer had a length of stay in ICU-B of 10.8 ± 14.0 days compared with patients after transfer of 8.0 ± 7.6 days. A total of 56 patients died in ICU-B, 26/160 (16.3%) in the period before transfer and 30/144 after transfer (20.8%). As shown in Figure 1, HAIs occurred in 31 of 304 patients (10.2%) during the study period, with no statistical difference between the two cumulative incidences (13.1% vs. 6.9%, *p* = 0.075). The total number of HAIs was 34, of which 18 (52.9%) were pneumonias, followed by ten bloodstream infections (29.4%) and five urinary tract infections (14.7%); a single case (2.9%) was reported as unspecified, one HAI occurred in 28 patients (90.3%), while the remaining three (9.7%) developed two HAIs.

## Discussion

This study found that there was no difference in the incidence of healthcare-associated infections in patients in ICU-B when the six-month period in the recovery room was compared with the 6 months period before unplanned transfer. The ICU-B mortality rate did not change after transfer, although hospital-wide data on ICU mortality rates were among the worse during the period studied.

Our results differed from the decrease in the incidence of HAIs reported by Ture et al. (19); in particular, these authors pointed out that the type of causative microorganisms and their susceptibility to antimicrobial agents, which we did not study, had not changed. This could be due to the better availability of hand washing facilities, hand sanitizers, and access points at the new site (i.e., posttransfer location), along with the added advantage of the new and clean environment that did not harbor already contaminated areas and fomites that could infect patients, offset by lower nurse-to-bed ratio, larger work area, and higher workload. The observation of a lower incidence of HAIs, which was also confirmed by Kim et al. (20), who found no changes in the detection of multidrug-resistant pathogens after an ICU move, seems somewhat consistent with our findings. As observed by colleagues (20), a shorter stay in the ICU could play an important role in such a reduction because patients are less exposed to the ICU. However, it is important to distinguish whether patients are transferred to a new location, i.e., a completely new ICU (which could be considered noncontaminated with respect to microorganisms) or to a unit previously used for other clinical activities (i.e., as in our case: recovery room within the operating room).

No adverse effects of ICU transfer on patient safety or quality of care were identified. The development of a set of structural, process, output/outcome indicators to monitor the impact of ICU transfers (urgent or planned) is desirable to standardize the process of identifying secondary sites, transferring units, and evaluating the impact of transfers. The indicators used in this study could be an initial proposal for further discussion at the national and international levels on the topic.

As outlined at WHO, retrospective analysis, which can also be referred to as after-action reviews (AARs), are extremely useful in leveraging best practices, identifying areas and actions for improvement, and promoting individual and collective learning (1). Indeed, this qualitative review is not about assessing individual performance or competencies, but rather about identifying functional challenges that need to be addressed, and best practices that should be maintained (1) from a Safety-II perspective (27).

Given all the potential threats, it remains critical for hospitals to develop a preparedness plan that covers as many emergency situations as possible (28, 29). These situations should include mass casualties resulting from natural events (e.g., hurricanes, tornadoes, wildfires, earthquakes), accidents (e.g., plane crashes, building collapses, toxic waste sites, nuclear events), and man-made crises (e.g., terrorism), pandemics, and wars. Many international organizations, such as the World Health Organization (WHO) (5), ECDC (30), and the Australasian College for Emergency Medicine (31), have developed documents and checklists to help managers of healthcare services develop their context-specific preparedness plan. Olivieri et al. have also developed the assessment tool, TIER that can be used in a standardized manner during simulated exercises and drills (32). The same issue has also been addressed at the European level through the development of a competency model for public health emergency preparedness (33). Nonetheless, the use of AAR offers the opportunity to enrich and better inform the preparedness and response cycle (1).

Preparedness and Plan B are essential for dealing with adverse events, but it is important to remember that hazards may also originate from within the hospital and affect only a portion of the building. Therefore, such contingency planning should always take into account the possible need to relocate one or more units (e.g., an intensive care unit, the emergency department, the neonatal intensive care unit, a highly specialized operating room) within the same hospital, so that hospital management may need to establish unused wards with different structural and technical equipment. Persoff et al. (28) have offered some thoughts along these lines, still citing the possibility of moving the entire damaged unit with all patients to another hospital. However, what if the damaged hospital is a highly specialized center that normally receives patients from other facilities? Further study and consideration is needed to better manage unit assignment and take more effective precautions against future threats.

Limitations: the results of this study should be considered in light of several limitations. First, we were unable to collect prospective data because the initial threat occurred unexpectedly during urgent ICU transfer to the recovery room. Therefore, our data were collected only retrospectively, which may have prevented us from examining organizational and clinical outcomes in more detail. In addition, the two sites were no longer available to the researchers at the time of the study, so it was not possible to assess the space around the beds (clear floor space) or the specific elements of the room layout (number of chairs, sharps containers, etc.). Second, we were not able to collect data on the working hours of the healthcare staff, which could have helped to take into account the different amount of time spent caring for each patient and to meet the organizational needs of the unit. Finally, with regard to reported adverse events, it should not be overlooked that the increased workload due to the adjustment to the new location may have resulted in less time to report adverse events in the transferred ICU, which may have been underreported.

In conclusion, in this analysis we sought to develop a set of structural, process, and outcome indicators to monitor the impact of ICU relocation and to standardize such an assessment. The set of indicators used in this study may be an initial suggestion for further discussion at the national and international levels, including for future benchmarking purposes.

Although the ICU transfer was a stressful time for all staff and the entire healthcare facility which had to readjust its organization, we did not observe any negative impact on patient outcomes in terms of clinical and organizational indicators. When the hospital faced this internal emergency in 2017, it was impossible to imagine that this was a dress rehearsal for what would come later with COVID-19. The unexpected impact of this flood on that January night showed hospital leadership once again that the uncertainty of the future can only be managed through the development and implementation of risk assessment and management procedures, as well as continuous professional development and performance improvement exercises.

## Data availability statement

The original contributions presented in the study are included in the article/supplementary material, further inquiries can be directed to the corresponding author.

## Ethics statement

The studies involving humans were approved by Regional Unique Ethics Committee (CEUR) of Friuli-Venezia Giulia. The studies were conducted in accordance with the local legislation and institutional requirements. The participants provided their written informed consent to participate in this study.

## Author contributions

LB: Conceptualization, Project administration, Writing – original draft, Investigation, Methodology. EM: Data curation, Investigation, Methodology, Writing – review & editing. MP: Conceptualization, Formal analysis, Investigation, Methodology, Writing – review & editing. DC: Conceptualization, Investigation, Methodology, Writing – review & editing. AM: Investigation, Methodology, Writing – review & editing. GB: Investigation, Methodology, Writing – review & editing. ADM: Writing – review & editing. CM: Writing – review & editing. RC: Supervision, Writing – review & editing. LL: Writing – review & editing. SB: Supervision, Writing – review & editing. LA: Conceptualization, Data curation, Formal analysis, Investigation, Methodology, Writing – original draft.
